# PheNetic: network-based interpretation of molecular profiling data

**DOI:** 10.1093/nar/gkv347

**Published:** 2015-04-15

**Authors:** Dries De Maeyer, Bram Weytjens, Joris Renkens, Luc De Raedt, Kathleen Marchal

**Affiliations:** 1 Dept. of Microbial and Molecular Systems, KULeuven, Leuven, 3000, Belgium; 2 Dept. of Information Technology (INTEC, iMINDS), U.Ghent, Ghent, 9052, Belgium; 3 Dept. of Computer Science, KULeuven, Leuven, 3000, Belgium; 4 Dept. of Plant Biotechnology and Bioinformatics, U.Ghent, Ghent, 9052, Belgium

## Abstract

Molecular profiling experiments have become standard in current wet-lab practices. Classically, enrichment analysis has been used to identify biological functions related to these experimental results. Combining molecular profiling results with the wealth of currently available interactomics data, however, offers the opportunity to identify the molecular mechanism behind an observed molecular phenotype. In this paper, we therefore introduce ‘PheNetic’, a user-friendly web server for inferring a sub-network based on probabilistic logical querying. PheNetic extracts from an interactome, the sub-network that best explains genes prioritized through a molecular profiling experiment. Depending on its run mode, PheNetic searches either for a regulatory mechanism that gave explains to the observed molecular phenotype or for the pathways (in)activated in the molecular phenotype. The web server provides access to a large number of interactomes, making sub-network inference readily applicable to a wide variety of organisms. The inferred sub-networks can be interactively visualized in the browser. PheNetic's method and use are illustrated using an example analysis of differential expression results of ampicillin treated *Escherichia coli* cells. The PheNetic web service is available at http://bioinformatics.intec.ugent.be/phenetic/.

## INTRODUCTION

Molecular profiling experiments, such as mRNA and/or protein expression measurements, provide direct information on which genes or gene products are (in)active under a certain condition. Statistical overrepresentation methods give quick functional insights into genes listed by those experiments, but fail to unveil how the genes from these lists are mechanistically related (1–3). Network based approaches (4,5) combine the vast amount of interactomics knowledge, represented as interaction networks, with the results of molecular profiling experiments to search for these mechanistic insights. Such integrative approaches have several benefits. First, the interaction networks help filtering noise from gene lists. Second, the interaction networks compensate for missing information: genes relevant to the process under study, but not in the gene list, can be recovered through their connectedness with the (in)active genes. Third, integrating multiple molecular levels into the interaction network (e.g. protein–protein, protein–DNA, phosphorylation, metabolic, …) provides a better insight into the process of interest.

Sub-network inference algorithms aim to reconstruct how genes from a gene list mechanistically interact (4,6). This is performed by inferring the sub-network from the interaction network that ‘best’ connects a set of listed genes, where ‘best’ depends on the biological question at hand. In this context, we have previously developed PheNetic, which uses probabilistic logical querying to infer sub-networks from omics-derived gene lists (7). PheNetic’ s performance in relation to the state-of-the-art and its biological relevance have been demonstrated through case studies (7,8).

State-of-the-art sub-network inference methods, despite relying on different computational methodologies (9–16), all have shown to be useful for omics data interpretation, each in their own specific application domain, e.g. to link genetic mutations to an expression phenotype, for gene prioritization, etc. However, because these methods are based on complex algorithms and workable implementations are often unavailable in the public domain, the practical usage of these methods is still limited. So far only few methods are accessible through an easy and intuitive web interface (17–19).

To offer a web service specifically tuned toward the analysis of gene lists identified from expression profiling experiments, we present PheNetic, which is wrapped around the similarly named core algorithm (7). Input data consist of an interaction network as a representation of the publicly available interactomics data (downloadable from the website for a large number of organisms), a differential expression data set and a list of genes of interest. PheNetic infers from the interaction network the sparsest sub-network that, based on the provided expression data set, is most likely differentially (in)activated between the compared conditions. The web service allows viewing and interpreting the resulting sub-networks in an interactive module. Additionally the inferred sub-networks can be downloaded in different formats for further analysis with tools such as Cytoscape (20). The PheNetic web service is free and open to all users without login requirement.

## METHODOLOGY

**Figure 1.**
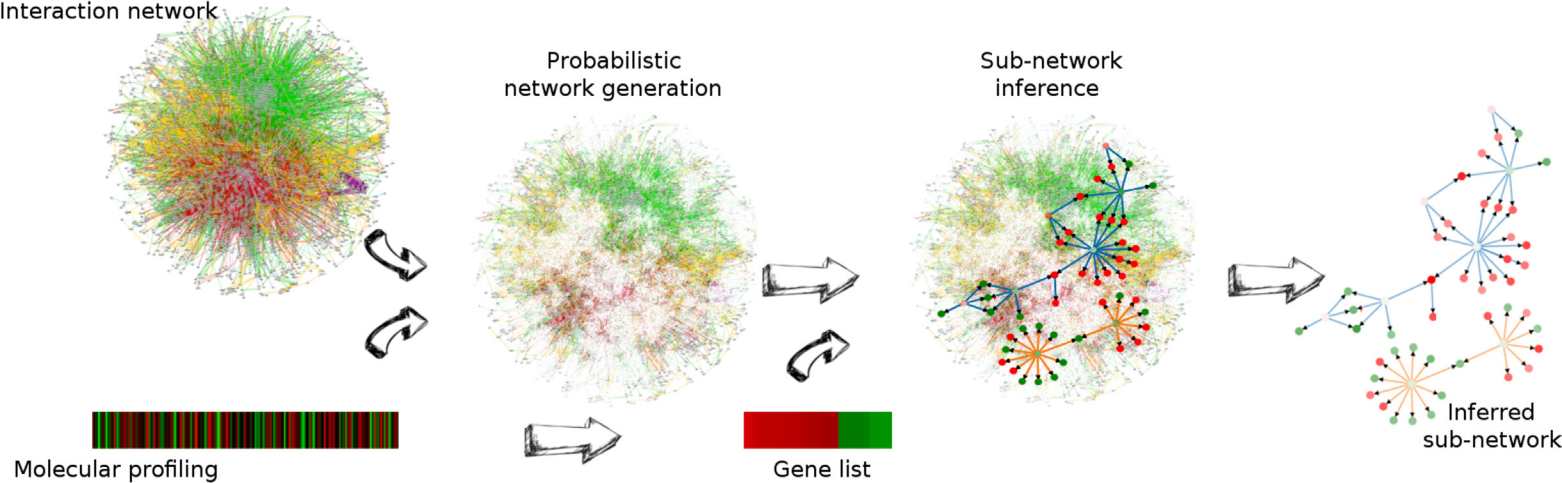
Overview of PheNetic, a web service for network-based interpretation of ‘omics’ data. The web service uses as input a genome wide interaction network for the organism of interest, a user generated molecular profiling data set and a gene list derived from these data. Interaction networks for a wide variety of organisms are readily available from the web server. Using the uploaded user-generated molecular data the interaction network is converted into a probabilistic network: edges receive a probability proportional to the levels measured for the terminal nodes in the molecular profiling data set. This probabilistic interaction network is used to infer the sub-network that best links the genes from the gene list. The inferred sub-network provides a trade-off between linking as many genes as possible from the gene list and selecting the least number of edges.

PheNetic exploits the vast amount of publicly available interactomics knowledge, represented as an interaction network to reason about likely mechanisms that drive a molecular phenotype, here reflected by a high-throughput differential expression experiment (Figure 1). Hereto, PheNetic selects from an interaction network ‘paths’ or ‘explanations’ of how the differentially expressed genes can be connected to each other. Based on these paths PheNetic then infers from the genome wide interaction network, a sub-network that connects as many as possible genes from the supplied gene list in the most parsimonious way i.e. using the least number of edges or using the smallest sub-network. It hereby assumes that genes from a gene list are involved in common pathways and thus that paths between these genes should ideally overlap. Depending on the run mode, PheNetic can focus on inferring either the upstream regulatory mechanisms that are causal to the observed differential expression phenotype or on the pathways/protein complexes that are (in)activated by the differentially expressed genes (Figure 1). PheNetic thus extracts from a genome wide static interaction network, the condition-dependent sub-network that is most likely activated or repressed under the assessed conditions.

**Figure 2.**
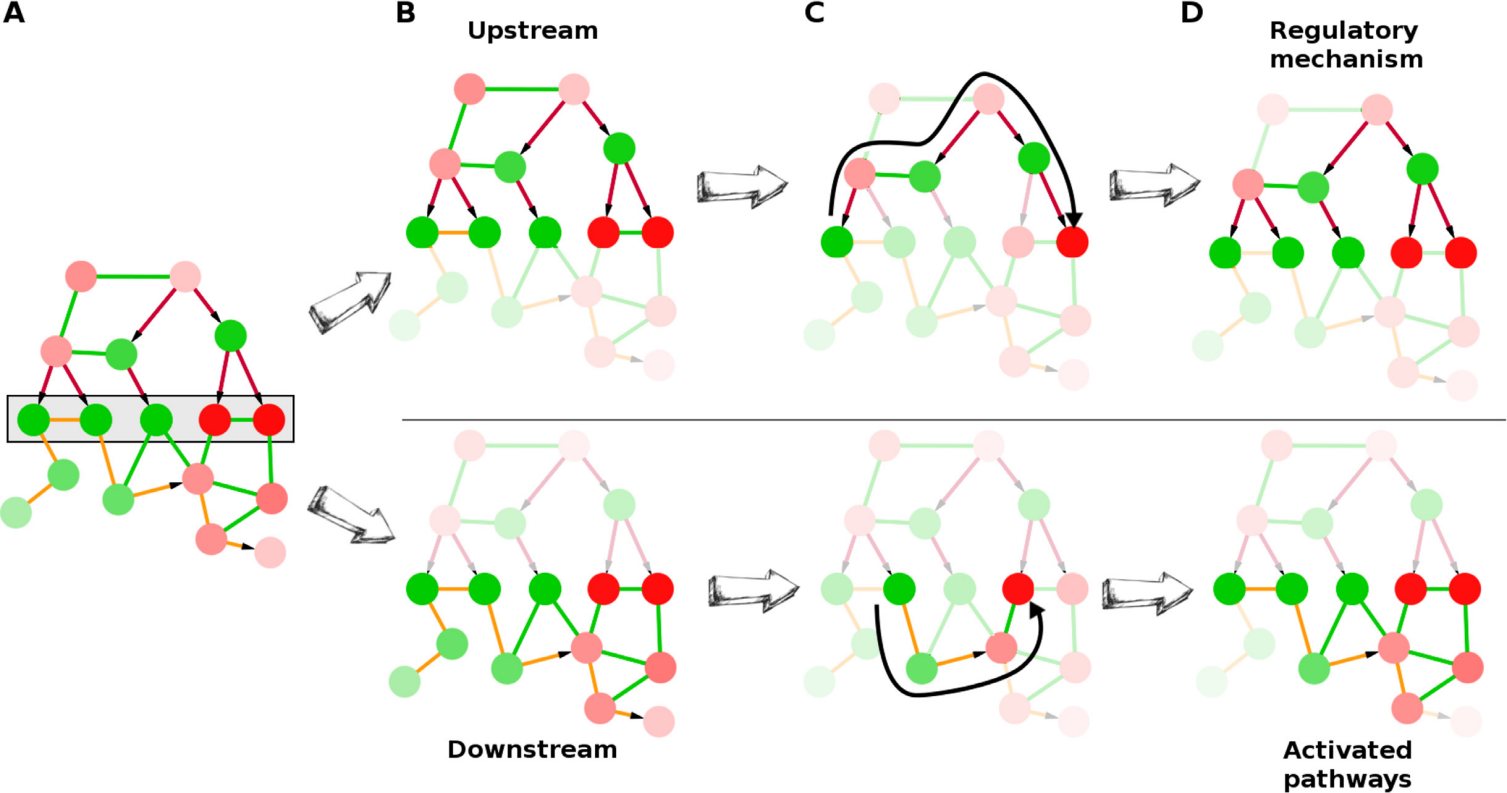
Conceptual representation of the sub-network inference by PheNetic. The colors of the edges indicate the different types of interactions with green referring to protein–protein, red to protein–DNA and orange to metabolic interactions. Arrows indicate the direction of the interaction. PheNetic will infer the sub-network from the interaction network that best connects the genes from a gene list (**A**, gray box), given the differential expression data. PheNetic can be used in two different run modes: the upstream run mode (**B** top) and the downstream run mode (**B**, bottom). To infer the upstream regulatory sub-network (**C**, top), paths (thick black arrow) between the genes of the gene list should first run upstream, against the natural direction of the interaction network, and then run downstream, following the natural direction of the interaction network. In addition to this, both the terminal edges of the path have to be regulatory interactions (e.g. DNA–Protein, sRNA, …). To infer the activated downstream pathways (**C**, bottom), paths between the genes of the gene list run downstream, hereby following the natural direction of the network. By selecting the smallest sub-network that best connects the genes from the gene list given the specific run mode, PheNetic is able to select the regulatory mechanisms responsible for the observed expression (**D**, top) or on the pathways/protein complexes that are differentially expressed or that result in the observed differential expression (**D**, bottom).

To solve the sub-network inference problem, PheNetic first uses the differential expression data to convert the genome wide interaction network *N* into a complete probabilistic network *F*, where *F* is simply *N* but with probabilities associated to the edges. The assumption here is that edges connecting differentially expressed genes have a higher probability to be (in)active under the studied conditions than edges between nodes that are not differentially expressed. This probabilistic interaction network now allows to assess the probability of connectedness *P*(*path*(*A*,*Y*)|*F*), i.e. the probability that there exists a path between *A* and *Y*. A path, in the context of this paper, is defined as a set of consecutive directed or undirected edges without cycles in the probabilistic network that connect start gene *A* from the gene list *L* to any other end gene *Y* from the gene list *L* and that are conform a given run mode. The probability of a path is simply the product of the probabilities of the edges along the path. PheNetic provides two different run modes (Figure 2). In the upstream run mode, the first and last edges of the path have to be regulatory interactions (e.g. DNA–Protein, sRNA, …). In addition, a path consists of a first part starting from the start gene, in which the path runs against the direction of the interaction network, i.e. against the direction of the edges when the edge is defined as directed, and a second part ending in the end gene, in which the path follows the direction of the network. By doing so the path describes a common regulatory mechanism for both the start and end node of the path. In the downstream run mode only paths that follow the direction of the network are valid.

The sub-network inference problem boils down to an optimization problem in which the ‘best’ sub-network *S*_optimal_ is selected. *S*_optimal_ corresponds to the highest scoring sub-network *S* according to Formula ((1)) and provides a trade-off between selecting the least number of edges and linking as many as possible genes from the gene list.

(1) \begin{equation*} O(S) = \sum\nolimits_{A \in L,\quad Y = L\backslash A} {P(path(A,Y)|S) - x_c *|S|} \end{equation*}

where *x_c_* is a constant cost factor. The last term imposes the sparsity of the inferred sub-network by penalizing linearly the sub-network size in number of edges with a factor *x_c_*. The first term assesses how well the genes from the list are connected in the inferred sub-network. As mentioned earlier, *P*(path(*A*,*Y*)|*F*) is the probability that gene *A* is connected to any gene *Y* from the gene list in the probabilistic network *F*. When selecting a sub-network *S*, this probability changes to *P*(path(*A*,*Y*)|*S*) as paths from *F* can become invalid in *S*, this because the sub-network contains less edges than the probabilistic network. Based on the score *O(S)* we can score each possible sub-network selected from the probabilistic network to infer *S*_optimal_. Inferring the probability *P*(path(*A*,*Y*)|*S*) is an NP-hard problem, that it is computationally hard to compute this exactly. Therefore, PheNetic approximates *P*(path(*A*,*Y*)|*S*) by rather than enumerating all paths that connect *A* to *Y*, restricting the number of valid paths to the k-best or k-most likely paths between gene *A* and any other gene *Y* from the gene list *L* in the complete probabilistic network (21). Knowledge compilation converts the approximation from the probabilistic network into a computationally tractable form (22). To obtain *S*_optimal_ a greedy hill climbing optimization is performed (7,23).

## INPUT

The input required by the web service consists of an interaction network of the organism under study, the differential expression data and a gene list.

### Interaction network

The interaction network is a comprehensive representation of all current interactomics knowledge on the organism of interest (4). Networks are represented as mixed graphs *G*(*N*,*E*) where nodes *N* correspond to biological entities (e.g. protein, RNA, gene, …) and edges *E* correspond to the interactions between the nodes (6,24). Every edge is assigned an edge type, indicating the molecular layer to which the interaction represented by the edge belongs to (e.g. protein–DNA interactions, protein–protein, …). Depending on its type and provided the proper information is available, an edge will be directed (e.g. protein–DNA interactions, sRNA, phosphorylation, …) or undirected (protein–protein interactions, undirected metabolic interactions, …).

The web service provides interaction networks for a large number of organisms. The provided interaction networks either correspond to manually curated networks used in previous publications (7,8) or to networks derived from the String database (25). Note that users can also upload their own networks without any constraint on the interaction types or network structure.

### Differential expression data set

To construct the probabilistic network *F* from the genome wide interaction network *N*, each edge is assigned a value that reflects how likely the start node and end node of the edge are (in)activated in the specific experimental condition given the differential expression data.

To this end, per node the probability that an expression value at least as extreme as the one associated with that node would be observed by chance is calculated given the null hypothesis that the gene which corresponds to the node is not significantly differentially expressed, is true. Calculation is performed using a two-tailed *T*-test assuming that the log fold changes follow a normal distribution *N*(*μ*,*σ*). By calculating the standard normal distribution *N*(0,1) of this normal distribution, the probability can be calculated for any differential expression value *D*_gene_ using Formula (2) in which *Z*_gene_ corresponds to the z-transformation of *D*_gene_.

(2) \begin{eqnarray*} \begin{array}{*{20}l} {P_{\hbox{\scriptsize\it gene}} = } \\ {\left\{ {\begin{array}{*{20}c} {{\kern-.6pt}P(X {>} Z_{\hbox{\scriptsize\it gene}} ) {+} P(X {<} {-}Z_{\hbox{\scriptsize\it gene}} )\,if\,Z_{\hbox{\scriptsize\it gene}} {>} 0} \\ {{\kern-.6pt}P(X {<} Z_{\hbox{\scriptsize\it gene}} ) {+} P(X >{-} Z_{\hbox{\scriptsize\it gene}} )\,if\,Z_{\hbox{\scriptsize\it gene}} {<} 0} \\ \end{array}} \right.} \\ \end{array}\nonumber \\ \end{eqnarray*}

As we are interested in giving high scores to genes which have high differential expression values, 1-*P*_gene_ will be used to score each gene. Using the cumulative normal distribution *Φ*(*μ*,*σ*) this can be simplified as shown in Formula (3). If no differential expression measurement for a specific gene is available, Score_gene_ is set to 0.5.

(3) \begin{equation*} Score_{gene} = abs(1 - 2*\Phi _{(\mu ,\sigma )} (D_{gene} )) \end{equation*}

These scores are subsequently used to define a measure for the probability that an edge is involved in a certain condition as the product of the scores of the genes at both ends of the interaction (Formula (4)).

(4) \begin{equation*} P_{edge} = Score_{edge\_start} *Score_{edge\_end} \end{equation*}

In terms of probabilistic networks *P*_edge_ denotes the probability that the edge is present, which also explains why the probability of a path is the product of the probability of the edges along that path.

We illustrate the effect of the probability calculation based on the sample data provided on the website. The log folds of the sample data have a mean of −0.036 and a standard deviation of 1.255. As an example the value for the edge between nhaA, with *D*_nhaA_ equal to −2.80, and nhaR, with *D*_nhaR_ equal to −2.00, is determined. As *Φ*(*μ*,*σ*)(*D*_nhaA_) is equal to 0.01 and *Φ*(*μ*,*σ*)(*D*_nhaR_) is equal to 0.05, the Score_nhaA_ is equal to 0.98 and the Score_nhaR_ is equal to 0.9 allowing to calculate *P*_edge_ equaling 0.88. This same exercise is performed for the edge between recA, with *D*_recA_ equal to 0.55, and narG, with *D*_narG_ equal to 5.17. Then *Φ*(*μ*,*σ*)(*D*_recA_) is equal to 0.60 and *Φ*(*μ*,*σ*)(*D*_narG_) is equal to 0.999 which means Score_recA_ equals 0.2 and Score_narG_ equals 0.998 resulting in *P*_edge_ equaling 0.199. This indicates that the edge between nhaA and nhaR receives a higher *P*_edge_ as both genes are clearly differentially expressed compared to the edge between recA and narG as recA only is slightly differentially expressed.

### Gene list

PheNetic will infer the sub-network from the interaction network that best connects the genes from a gene list, given the differential expression data. The most straightforward way of defining a gene list is to select from the differential expression data set the most differentially expressed genes based on log fold changes and/or *P*-values. However, the user is free to provide any list of genes. For example, a list of genes filtered based on criteria different than those offered by the web service and/or a list of genes for which the user wants to know whether they are related to the pathways triggered by the differential expression data set but that are not necessarily differentially measured themselves.

### Parameters

When starting an analysis the user has to specify the run mode. Two run modes are available, namely the upstream mode, to infer the gene regulatory network acting upstream of the expression response and, the downstream mode to infer the (in)activated pathways. Additionally, the user has to specify the cost (see Formula (1)). Decreasing the cost increases the size of the inferred sub-network and vice versa. By stepwise decreasing the cost, the user will find an ordered series of sub-networks starting with the smallest sub-network containing the least number of edges that best link the genes in the gene list and then gradually obtaining larger networks.

Additionally the user can change more advanced parameters such as the path length and the k-best paths. The path length specifies the length of the ‘paths’ or ‘explanations’ that connect the genes from the gene list through the interaction network. The range of the path length is fixed between 2 and 5 interactions, based on both biological (26–28) and computational considerations. By default the path length is set to 4 based on the results of the original PheNetic publication (7). The ‘k-best paths’ parameter indicates how many of the most likely paths between gene *A* and any gene *Y* from the gene list PheNetic should use to approximate the probability of connectedness between *A* and *Y*. The selection of the k-best paths and their probability defines the size of the search space from which the most optimal sub-network will be computed. Higher values for k means sampling a larger search space and a potentially more optimal selected sub-network, but this comes at the expense of longer running times. The parameter can be set between 5 and 50.

## OUTPUT

**Figure 3.**
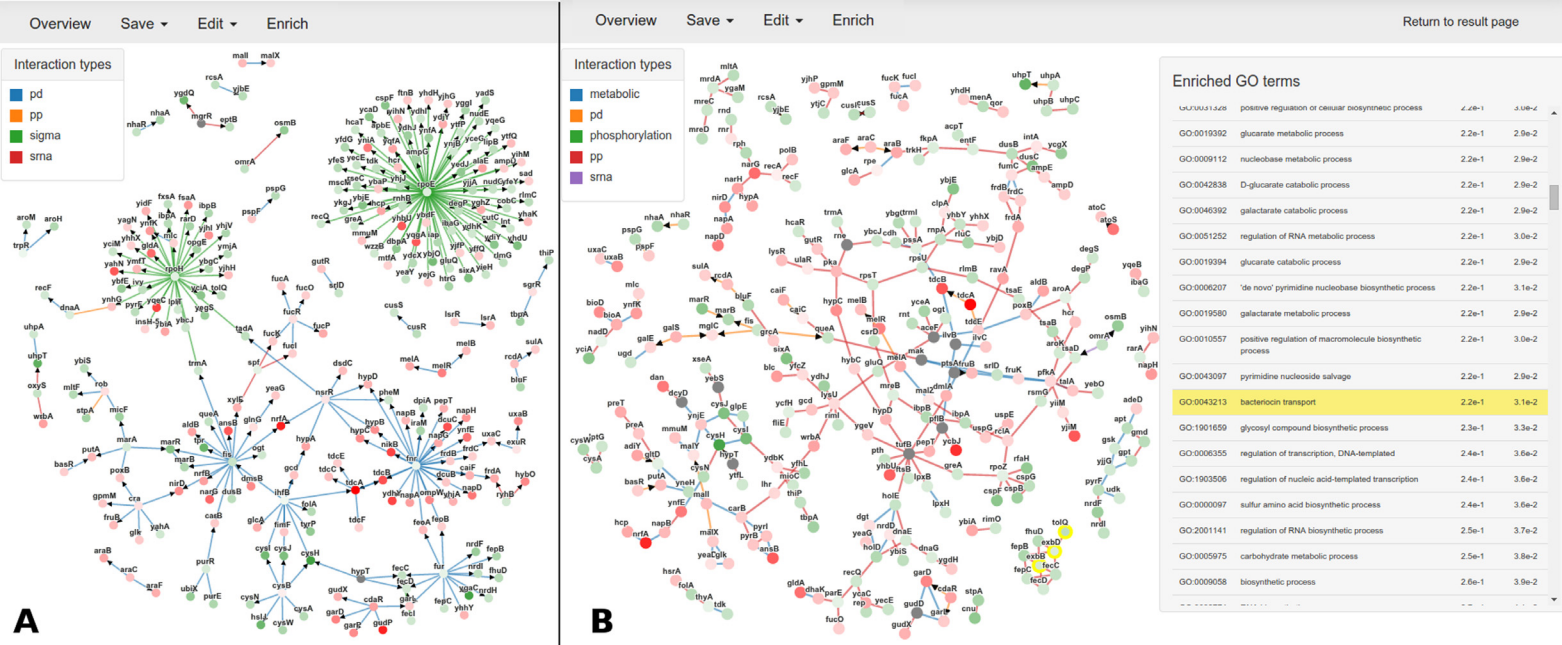
Representative result of PheNetic on the test data set, measuring the differential expression behavior of *E. coli* cells subjected to Ampicillin. (**A**) Upstream run mode. This mode recovers the regulatory mechanism identifying regulators potentiating the observed differential expression,such as the pleiotropic global regulator Fis, the respiratory regulators FNR and NsrR, the regulator of iron homeostasis Fur, the stationary phase sigma factor rpoH and the ROS mediated response regulators OxyS/RpoE (**B**) Downstream run mode. This mode recovers differentially activated/repressed pathways such as the nitrate metabolism, iron ion homeostasis and anaerobic respiration. In the network visualization, the level of differential expression of the nodes is indicated by red and green for respectively over and under-expression. The more intense the color, the higher the level of differential expression. The color of the edge indicates the interaction type. If an interaction is directed according to its original interaction source, this is indicated by an arrow.

On job completion the inferred sub-network can easily be displayed by loading the results in the visualization module (Figure 3). An interactive network is visualized in the browser which shows the biological entities and their interactions. The differential expression levels are represented by the color of the nodes where green refers to under- and red to over-expression. The color of an edge indicates the interaction type and the arrow, if applicable, indicates the direction of the edge. The visualization module allows users to further annotate and explore the inferred sub-network by providing the possibility to upload standard gene names and to perform a GO enrichment test. To perform gene enrichments, the user has to upload an annotation file in the format as defined by Gene Ontology (29). Genes associated with each of the enriched GO terms will be highlighted in the visualized sub-network, upon clicking the corresponding enriched term. This allows the user to quickly identify clusters of similar functionality in the sub-network. To capture the annotated sub-network, snapshots can be taken inside the browser. Inferred sub-networks can be downloaded in multiple formats, compatible with other network visualization tools such as the SIF format for Cytoscape (20).

## USE CASE

To illustrate a typical workflow, an example analysis on a publicly available data set was performed (Gene Expression Omnibus, GSE56133), measuring in *Escherichia coli* the effect of ampicillin on expression behavior (30). The example data can be loaded in the web service by clicking the load example buttons or can be downloaded from the help pages. The gene list is generated by selecting the genes with a log fold change above 1.5 in combination with a corrected *P*-value below 0.05.

First, PheNetic was used to infer the upstream regulatory program (Figure 3A), driving the observed differential expression. To this end, the upstream run mode is selected in combination with default parameter settings. The analysis connects 544 genes on an interaction network containing more than 18731 interactions in under a minute. Zooming in on the resulting sub-network reveals the inferred regulatory program which contains the pleiotropic global regulator Fis, the respiratory regulators FNR and NsrR, involved in respectively anaerobiosis and cell protection against nitric oxide (NO), Fur known to be involved in iron homeostasis, rpoH responsible for stationary phase response and finally OxyS/RpoE involved in an ROS mediated response. These observations correspond to the biology of the experiment, which hypotheses that ‘addition of antibiotics’ interferes with the bacterial physiology through the generation of reactive oxygen species that are known to induce pleiotropic effects by means of general stress response regulators (Fur, rpoH-rpoE, oxyS) (30). Although an antibiotic mediated induction of rpoH and FNR cannot be excluded the presence of these regulators in the sub-network could also be related to the general physiological state of the cells (stationary phase transition toward micro-aerobiosis). These results illustrate how the resulting sub-network can help prioritizing plausible regulators of the observed molecular phenotype. Moreover, many of the regulatory genes that do not themselves display high levels of differential expression can be recovered in the inferred sub-network because of their connectedness with significantly differentially expressed genes (e.g. FNR, cysB, Fur and rpoE).

To identify the pathways triggered by the differentially expressed genes, we run PheNetic in the ‘downstream’ run mode, in combination with default parameter settings. Figure 3B shows how the resulting sub-network is different from the one selected with the upstream run mode. In contrast to the latter one which is sparser and more focused on regulators linked to the differentially expressed genes, the network identified with the downstream run mode consists of strongly connected components and ‘linear’ pathways. These components mostly contain genes with similar functionalities or involved in the same pathways that are together differentially up or down regulated. From these results it is possible to identify activated pathways/protein complexes associated with mechanisms such as anaerobic respiration, iron homeostasis, carbohydrate metabolism, … with the help of the provided enrichment tool.

## CONCLUSION

Viewing in house generated gene lists in the light of the growing amount of interactomics knowledge will become mandatory: integrating one's own experimental results with these complementary resources allows for a more robust analysis and a more global view on the molecular mechanism. Web servers such as Responsenet2.0 (19), SteinerNet (17) and PheNetic anticipate on this increasing need for integrative analysis by providing non-expert users access to non-trivial sub-network inference methods and allowing them to view their own in-house data in the light of current interactomics knowledge. PheNetic provides an automated flow in which an uploaded gene list is interpreted using precompiled interactomics networks. Depending on the run mode, users can focus on extracting the sub-network from the interaction network that drives (upstream regulatory analysis) or is reflected by the observed expression phenotype (downstream analysis).

The main difference between PheNetic and the already available web servers ResponseNet and SteinerNet is the underlying algorithmic approach which determines the particularities of the selected sub-networks as well as their intended applications. ResponseNet is a flow based algorithm that infers the subnetwork that best connects sources to targets over the interaction network. This type of analysis makes ResponseNet suitable for analyzing cause-effect data such as the analysis of knock-out screenings in combination with transcriptomics data. SteinerNet infers Steiner trees, or minimum spanning trees that connect sets of genes in the most optimal way over the interaction network. As this method selects a tree structure from the interaction network, sub-networks selected by SteinerNet cannot contain parallel paths between the selected genes, in contrast to the sub-networks detected by PheNetic.

All three web servers interpret in-house data using interaction networks: SteinerNet and PheNetic can be used to interpret differential expression data and ResponseNet to interpret cause effect data. The web servers provide modules to visualize and interpret the obtained sub-networks in an interactive environment. The SteinerNet interface provides the data to be downloaded and analyzed in network tools such as Cytoscape, whereas the interface of ResponseNet allows for a more elaborate analysis providing the editing of the selected network and an exploratory analysis of the genes selected in the resulting sub-network. Both web servers focus on analyzing data from human, other vertebrate model organisms and yeast, providing networks for those organisms only. PheNetic specifically focuses on the analysis of expression profiling experiments, hereby providing networks for a wide variety of organisms, with a focus on micro-organisms (bacteria and yeast). As it is non-trivial in the context of sub-network inference to statistically assess the significance of the predictions, the available web servers provide summarizing statistics and/or GO enrichment analysis of the inferred sub-networks as additional validation steps.
